# Game based physiotherapy for evidence based practice in children with juvenile idiopathic scoliosis

**DOI:** 10.1186/1748-7161-8-S1-O16

**Published:** 2013-06-03

**Authors:** P Feistritzer-Gröbl, A Nischelwitzer, V Saraph

**Affiliations:** 1 University of Applied Sciences FH JOANNEUM, Graz, Austria; 2 Department of Pediatric Surgery Medical University of Graz, Graz, Austria

## Background

The performance of daily exercises of patients with “juvenile idiopathic scoliosis” (JIS) during the home-based exercise period is difficult to monitor; therefore the collection of evidence is a challenge.

## Aim

The aim of this study was to use a specialised computer programme to guide, monitor and evaluate therapeutic exercises. With this programme, exercises are monitored so that they are carried out in a precise way, and children get a real-time feedback. Furthermore, data is recorded for further evaluation by the therapist.

## Methods

Software and interfaces of a 3D input device were used to detect and measure smallest movements within a game scenario. To maintain motivation three different games were used. The study group were 20 children between 9 and 13 years old, with the diagnosis JIS (Cobb curve of 23° +/- 4°, bracing with Chêneau-braces). The children exercised six months according to the Schroth concept; two standardized exercises of this concept were carried out guided by the computer program. Data was recorded for each patient regarding exercises time and scores, exercise fault rates, stability, and mobility scores and self-efficacy scores. Correlations were calculated between different variables.

## Results

Considering the changes of faults over time, it can be seen that the variable date had a highly-significant negative influence on mistakes in the x, y and z- direction. (P-value: 0.004978)

## Conclusions

The available computer program for patients with JIS is suitable for evaluating therapeutic exercises. The collected quantitative data gives valuable information on the exercise regime, and can be used to monitor and evaluate treatment process. Due to the monitoring of the precise performance of exercises, exercise times are used more efficiently. A randomized study with the same target group is currently being carried out to evaluate possibly different results of treatment with, or without, a computer game.
